# Effect modification of gallbladder wall thickness on the association between fibrosis-4 index and high-risk colorectal adenoma in patients with gallbladder polyps: a retrospective cross-sectional study

**DOI:** 10.3389/fonc.2026.1972315

**Published:** 2026-10-07

**Authors:** Wenting Xu, Shuang Chen, Lihong Wang

**Affiliations:** 1 Department of Gastroenterology, Zhengzhou Central Hospital Affiliated to Zhengzhou University, Zhengzhou, China; 2 Medical Key Laboratory for Diagnosis and Treatment of Colorectal Cancer in Henan Province, Zhengzhou, China; 3 Zhengzhou Key Laboratory for Diagnosis, Treatment and Research of Colorectal Cancer, Zhengzhou, China

**Keywords:** fibrosis-4 index, gallbladder polyp, gut-liver axis, high-risk colorectal adenoma, metabolic syndrome

## Abstract

**Background:**

Gallbladder polyps (GBPs) and colorectal neoplasia frequently coexist against a background of metabolic dysfunction. This study aimed to investigate the association between the Fibrosis-4 (FIB-4) index and high-risk colorectal adenoma (HRA) in patients with GBPs, and to evaluate the effect modification of gallbladder ultrasound characteristics on this association.

**Methods:**

This retrospective study included 620 patients with GBPs who underwent colonoscopy at Zhengzhou Central Hospital Affiliated to Zhengzhou University between 2022 and 2025. Patients were classified into no adenoma, non-high-risk adenoma (non-HRA), and HRA groups based on colonoscopic pathology. The Mantel-Haenszel linear trend test was used to assess the dose-response relationship between FIB-4 category and adenoma severity. Multivariable logistic regression was fitted with HRA as the outcome, adjusting for demographic and metabolic covariates. Sensitivity analysis was performed using FIB-4 as a continuous variable (per standard deviation increment). Prespecified subgroup analyses and multiplicative interaction models were conducted, with Wald and likelihood ratio tests (LRTs) evaluating the effect modification of gallbladder wall thickness on the FIB-4-HRA association.

**Results:**

The FIB-4 category showed a dose-response relationship with adenoma severity (trend P = 0.0039). The risk of HRA in the high FIB-4 group was elevated in the unadjusted model (OR 5.83, P < 0.001) but attenuated after adjustment (aOR 2.32, P = 0.129). The overall aOR per 1-standard deviation (SD) increase in FIB-4 was 1.09 (P = 0.41). Subgroup analysis revealed that the association between per-1-SD increase in FIB-4 and HRA was present only among patients with gallbladder wall thickening (≥3 mm) (aOR = 6.38, P = 0.003), with no significant association in those with normal wall thickness (aOR = 0.94, P = 0.75). The multiplicative interaction term between FIB-4 and gallbladder wall thickness was significant (Wald P = 0.008, LRT P = 0.001).

**Conclusion:**

Subgroup analyses of this study showed that among patients with GBPs and gallbladder wall thickening, elevated FIB-4 was associated with an increased risk of high-risk colorectal adenoma. This association still requires validation in independent external cohorts.

## 1 Introduction

Colorectal cancer (CRC) is the second leading cause of cancer-related mortality worldwide (1). The vast majority of sporadic CRCs arise through the adenoma-carcinoma sequence, and early detection and removal of colorectal adenomas, particularly high-risk adenomas (HRAs), are critical for CRC prevention (2).

Gallbladder polyps (GBPs) are among the most common incidental findings on abdominal ultrasound; however, current clinical management primarily focuses on assessing their intrinsic malignant potential based on polyp size, number, and gallbladder wall characteristics (3). Nevertheless, the clinical significance of GBPs may extend beyond localized disease. A meta-analysis encompassing 20 studies demonstrated that gallbladder disease is associated with an increased risk of colorectal neoplasia, with patients with GBPs, especially larger polyps, carrying a higher risk of colorectal tumors (4), suggesting that these patients may represent a population at elevated risk for colorectal neoplasia.

The pathological basis linking GBPs and colorectal neoplasia has not been fully elucidated, but both conditions are associated with metabolic dysfunction. Metabolic dysfunction-associated steatotic liver disease (MASLD), as the hepatic manifestation of metabolic dysfunction, links the two: accumulation of MASLD components (≥4 components) is associated with an increased risk of GBP development (5), and a meta-analysis of 29 studies has also confirmed that MASLD is associated with an elevated risk of colorectal neoplasia (6). This association may be mediated by the gut-liver axis: metabolic dysfunction induces chronic low-grade inflammation, alterations in the bile acid profile, and gut microbiota dysbiosis, thereby promoting colorectal mucosal lesions (7–9).

The FIB-4 index, valued for its simplicity and reproducibility, was initially used for noninvasive stratification of liver fibrosis in chronic liver disease, and its application has expanded in recent years to risk assessment and prognostic prediction of extrahepatic malignancies. In patients with MASLD, more advanced liver fibrosis assessed by FIB-4 is associated with a higher risk of advanced colorectal neoplasia, including advanced adenoma (10). In patients following curative resection for CRC and those undergoing hepatectomy for colorectal liver metastases, elevated FIB-4 similarly indicates poorer survival outcomes (11, 12).

However, the aforementioned evidence regarding FIB-4 and colorectal neoplasia risk assessment and prognosis is largely derived from patients with MASLD or CRC, and has not been validated in the potentially high-risk subgroup of patients with GBPs. Given the shared metabolic dysfunction and gut-liver axis underpinnings, if this noninvasive index can also reflect the risk of colorectal adenoma in this population, it may provide a novel strategy for noninvasive identification of high-risk subgroups requiring colonoscopy, thereby shifting CRC prevention to the precancerous stage. Therefore, this study aimed to investigate the relationship between the FIB-4 index and HRA in patients with GBPs, and to analyze the effect modification of gallbladder ultrasound characteristics on this association.

## 2 Materials and methods

### 2.1 Study population

This retrospective study included clinical data from 620 patients diagnosed with GBPs by abdominal ultrasound at Zhengzhou Central Hospital Affiliated to Zhengzhou University between 2022 and 2025, who underwent colonoscopy within 1 month. The indications for colonoscopy in this cohort comprised opportunistic examination (performed during visits for asymptomatic conditions or non-gastrointestinal complaints), non-alarm gastrointestinal symptoms, and alarm symptoms; systematic screening was not conducted. Specifically, 350 patients (56.5%) were asymptomatic or presented for non-gastrointestinal complaints, and their colonoscopy was performed opportunistically during visits for GBPs or during hospitalization for other systemic diseases; 235 patients (37.9%) presented with non-alarm gastrointestinal symptoms such as abdominal pain, bloating, and acid reflux; and only 35 patients (5.6%) underwent colonoscopy because of alarm symptoms such as weight loss and hematochezia. Inclusion criteria included: age ≥18 years; complete clinical records. Exclusion criteria included: missing important laboratory data or pathology results; incomplete colonoscopy due to inadequate bowel preparation or other reasons; history of colorectal disease, including prior polypectomy, inflammatory bowel disease, familial adenomatous polyposis, melanosis coli, or ischemic colitis; patients with gallbladder polyps concurrent with gallstones; severe cardiac, pulmonary, hepatic, or renal dysfunction. This study was approved by the Ethics Committee of Zhengzhou Central Hospital Affiliated to Zhengzhou University (Approval No. ZXYY2026036). Given its retrospective nature, the Ethics Committee waived the requirement for written informed consent.

### 2.2 Diagnostic criteria and variable definitions

The FIB-4 index was calculated using the following formula: $\left[\text{age }(\text{years})\;\times \text{ aspartate transaminase level }(\text{U}/\text{L})]/[\text{platelet count }(10^{9}/\text{L})\times \sqrt{\text{alanine aminotransferase level }(\text{U}/\text{L})}\right]$, and was categorized according to standard clinical thresholds (13): low (<1.3), intermediate (1.3–2.67), and high (>2.67). This index was originally proposed by Sterling et al. (14).

In accordance with the 2020 consensus of the US Multi-Society Task Force on Colorectal Cancer (15), HRA was defined as the presence of advanced adenoma (i.e., diameter ≥10 mm, or containing villous histological components, or with high-grade intraepithelial neoplasia) or detection of ≥3 adenomas in a single colonoscopy. Patients were classified into three groups based on colonoscopic pathology: no adenoma, non-high-risk adenoma (non-HRA), and HRA. In addition, according to clinical ultrasound imaging criteria (16), the normal gallbladder wall thickness is <3 mm in the fasting state, and gallbladder wall thickening is defined as a wall thickness ≥3 mm.

### 2.3 Metabolic burden score

The metabolic burden score was constructed based on the component framework of the metabolic syndrome diagnostic criteria jointly issued in 2009 by the International Diabetes Federation (IDF), American Heart Association (AHA), National Heart, Lung, and Blood Institute (NHLBI), and other societies (17). Given the retrospective nature of this study, the lack of uniform waist circumference measurements, and the fact that Asian populations exhibit significant visceral fat accumulation and metabolic abnormalities at lower waist circumference levels, hepatic fat content may reflect insulin resistance more directly than waist circumference (18). Therefore, this study used fatty liver diagnosed by abdominal ultrasound as a surrogate for waist circumference as the central obesity indicator. The metabolic syndrome framework lists both elevated triglycerides and reduced HDL-C as components, which together reflect atherogenic dyslipidemia and underlying insulin resistance. In our simplified score, we merged the two into a single dyslipidemia component, defined as elevated triglycerides (TG ≥1.70 mmol/L) or reduced high-density lipoprotein cholesterol (HDL-C: <1.04 mmol/L for men, <1.3 mmol/L for women), with either abnormality counting as 1 point. The score incorporated 4 metabolic abnormality components, each scoring 1 point, for a total of 0–4 points: (1) fatty liver; (2) dyslipidemia; (3) hypertension (blood pressure ≥130/85 mmHg, or previously diagnosed hypertension, including use of antihypertensive medications); (4) dysglycemia (fasting plasma glucose [FPG] ≥5.6 mmol/L, or previously diagnosed diabetes, including use of hypoglycemic medications). It should be noted that this score is a non-standard, equally weighted composite indicator simplified from the standard metabolic syndrome framework, and it is primarily used as a covariate to adjust for metabolic confounding. Unlike the formal metabolic syndrome definition, which requires at least 3 of 5 criteria to be met, the component weights and scoring method of this score have not been validated by independent data.

### 2.4 Data collection

Clinical data were retrospectively collected from electronic medical records, including the following variables: demographic and metabolic covariates, including age, sex, body mass index (BMI), smoking, alcohol consumption, hypertension, dysglycemia, dyslipidemia, fatty liver, and a composite metabolic burden score (range 0–4: hypertension, dysglycemia, dyslipidemia, and fatty liver each scoring 1 point); laboratory indices including aspartate aminotransferase (AST), alanine aminotransferase (ALT), gamma-glutamyl transferase (GGT), and platelet count (PLT); the FIB-4 index calculated from age, AST, ALT, and PLT; gallbladder ultrasound characteristics including GBP number, gallbladder wall thickness (≥3 mm or <3 mm), and maximum GBP diameter; and colonoscopic pathology results.

### 2.5 Ultrasound examination and colonoscopy quality assessment

All patients underwent abdominal color Doppler ultrasound examination in the fasting state, which was performed as part of routine clinical care in the hospital’s ultrasound department. Gallbladder wall thickness was measured by imaging physicians with diagnostic ultrasound qualifications using real-time two-dimensional ultrasound during routine examinations. On long-axis and short-axis sections of the anterior gallbladder wall, an electronic caliper was used to measure from the hyperechoic line of the mucosal layer to the outer serosal margin, and the maximum wall thickness in the fasting, distended state was recorded; gallbladder wall thickening was defined as a wall thickness ≥3 mm. Given the retrospective design, ultrasound reports were retrospectively extracted from the hospital’s ultrasound information system, and study team members did not re-measure the images. Colonoscopy was performed by qualified digestive endoscopists at the Digestive Endoscopy Center following routine clinical protocols. In this study, colonoscopy quality indicators were retrospectively extracted from the endoscopy reporting system and verified: all included colonoscopies had a withdrawal time ≥6 min, bowel preparation quality was assessed using the Boston Bowel Preparation Scale with a total score ≥6, and all achieved complete colonoscopy (reaching the cecum).

### 2.6 Statistical analysis

Normally distributed continuous variables are expressed as mean ± standard deviation (SD), non-normally distributed continuous variables as median (Q1, Q3), and categorical variables as frequency (%). Patients were classified into no adenoma, non-HRA, and HRA groups based on colonoscopic pathology. Group comparisons used one-way analysis of variance (ANOVA) for normally distributed continuous variables, the Kruskal-Wallis H test for non-normally distributed variables, and the chi-square test for categorical variables (Fisher’s exact test was used when expected frequencies were <5). FIB-4 was categorized into low, intermediate, and high groups based on clinical cutoffs (<1.3, 1.3-2.67, >2.67), and its association with the ordinal outcome (no adenoma < non-HRA < HRA) was assessed using the Mantel-Haenszel linear trend test. Binary logistic regression was fitted with HRA as the outcome, adjusting for age, sex, smoking, alcohol consumption, and metabolic burden score. Sensitivity analysis was performed using FIB-4 as a continuous variable per 1-SD increase. Prespecified subgroup analyses evaluated the effect modification of sex, age, metabolic burden, GBP number, and gallbladder wall thickness on the FIB-4-HRA association; subgroup models were fitted within the corresponding strata and adjusted for remaining covariates. For gallbladder wall thickness, a multiplicative interaction model was additionally fitted, with the Wald test and likelihood ratio test (LRT) used for evaluation. The above subgroup analyses and multiplicative interaction tests were prespecified before data analysis based on prior literature and biological plausibility, rather than being selected *post hoc* according to the observed results of this study. Given that the number of HRA events was small (n = 9) within the gallbladder wall thickening subgroup (n = 90), the event-per-variable ratio in this subgroup was low, posing a risk of model overfitting. Accordingly, robustness analyses were performed using Firth penalized logistic regression and a simplified model adjusting only for age and sex. All tests were two-sided, with P < 0.05 considered statistically significant. All statistical analyses were performed using R version 4.6.1.

## 3 Results

### 3.1 Baseline characteristics of the study population

A total of 620 patients with GBPs were ultimately included in this study; the participant selection flowchart is shown in **Figure 1**. There were 340 males (54.8%) and 280 females (45.2%), with a mean age of 50.5 ± 11.0 years. Based on colonoscopic pathology, patients were classified into the no adenoma group (n = 433, 69.8%), non-HRA group (n = 110, 17.7%), and HRA group (n = 77, 12.4%). Baseline characteristics are presented in **Table 1A**. The distribution of the individual HRA component criteria is presented in **Table 1B**: among the 77 patients with HRA, 53 (68.8%) had ≥3 adenomas, 29 (37.7%) had villous histological components, 27 (35.1%) had adenoma diameter ≥10 mm, and 0 (0.0%) had high-grade intraepithelial neoplasia. There were statistically significant differences among the three groups in age, male proportion, metabolic burden score (≥2), hypertension, dyslipidemia, smoking history, FIB-4 index, ALT, and GGT (P < 0.05). Age, male proportion, proportion of metabolic burden score ≥2, FIB-4 index, and GGT levels generally increased with adenoma severity, and the prevalence of hypertension, dyslipidemia, and smoking history also increased correspondingly, all being highest in the HRA group. No statistically significant differences were observed among groups in BMI, AST, PLT, alcohol consumption, dysglycemia, fatty liver, maximum GBP diameter, gallbladder wall thickness, or GBP number.

**Figure 1:**
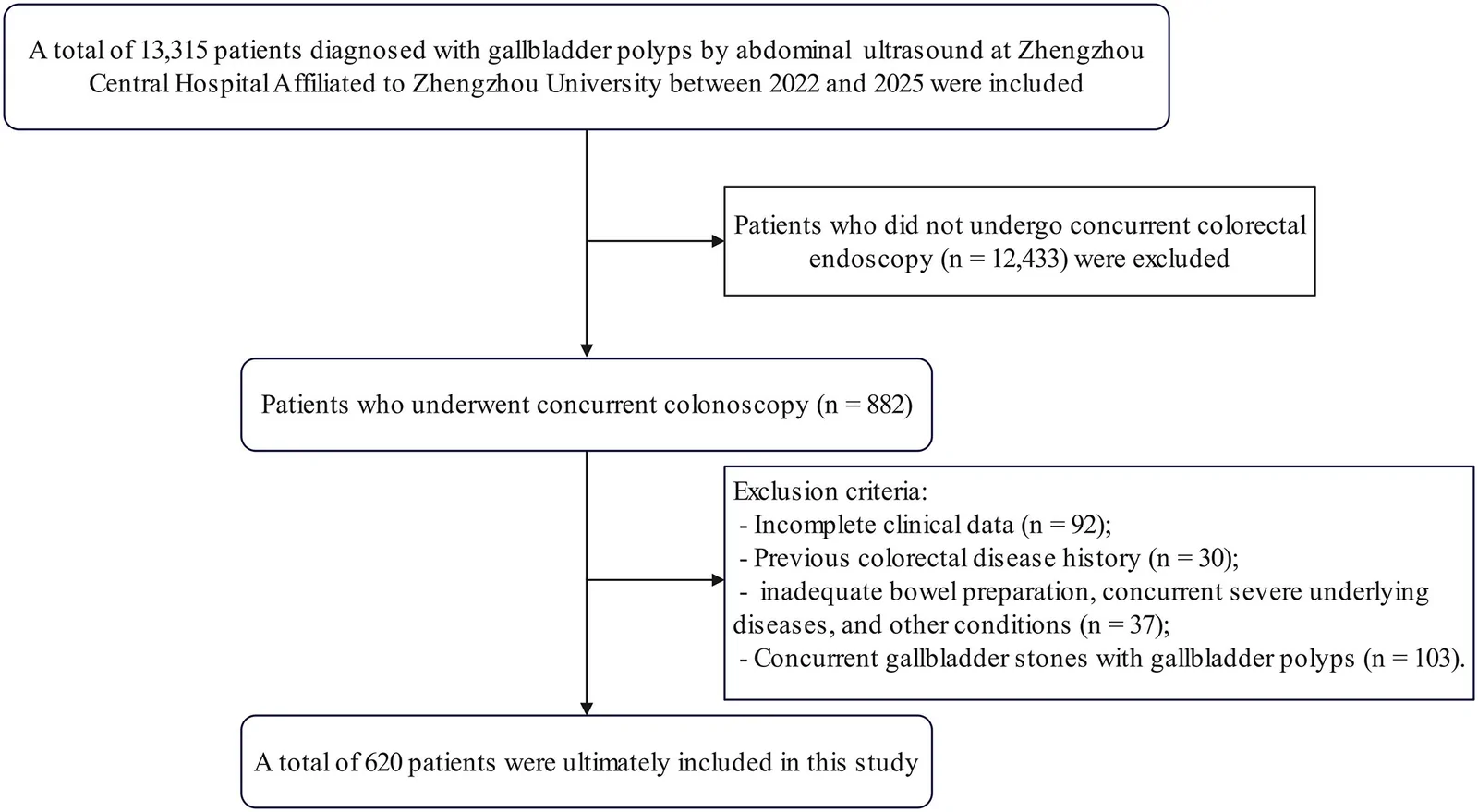
Participant selection flowchart.

**Table 1A:** Baseline characteristics of the study population.

Variables	No adenoma (n=433)	Non-HRA (n=110)	HRA (n=77)	P value
Age (years)	49.2 ± 10.4	52.1 ± 11.5	56.0 ± 11.1	<0.001
BMI (kg/m²)	24.2 ± 3.0	24.6 ± 3.1	24.9 ± 3.2	0.068
FIB-4 index	1.0 (0.8, 1.4)	1.2 (0.8, 1.4)	1.2 (0.9, 1.7)	0.012
AST (U/L)	20.0 (17.0, 25.0)	20.0 (17.0, 25.0)	23.0 (17.0, 31.0)	0.065
ALT (U/L)	19.0 (14.0, 29.0)	18.0 (15.0, 27.5)	23.0 (15.0, 34.0)	0.037
GGT (U/L)	20.0 (14.0, 37.0)	21.0 (15.0, 32.5)	24.0 (19.0, 36.0)	0.044
PLT (10^9^/L)	226.0 (195.0, 265.0)	228.0 (196.0, 262.8)	217.0 (182.0, 263.0)	0.617
GBP maximum diameter (mm)	4.0 (3.0, 5.1)	4.0 (3.2, 5.0)	3.9 (3.0, 4.6)	0.256
Sex, n (%)				0.003
Male	221 (51.0)	64 (58.2)	55 (71.4)	
Female	212 (49.0)	46 (41.8)	22 (28.6)	
Smoking, n (%)				0.031
No	401 (92.6)	96 (87.3)	65 (84.4)	
Yes	32 (7.4)	14 (12.7)	12 (15.6)	
Alcohol drinking, n (%)				0.321
No	408 (94.2)	100 (90.9)	70 (90.9)	
Yes	25 (5.8)	10 (9.1)	7 (9.1)	
Hypertension, n (%)				0.020
No	341 (78.8)	77 (70.0)	51 (66.2)	
Yes	92 (21.2)	33 (30.0)	26 (33.8)	
Dysglycemia, n (%)				0.088
No	191 (44.1)	43 (39.1)	24 (31.2)	
Yes	242 (55.9)	67 (60.9)	53 (68.8)	
Dyslipidemia, n (%)				0.043
No	205 (47.3)	43 (39.1)	26 (33.8)	
Yes	228 (52.7)	67 (60.9)	51 (66.2)	
Fatty liver, n (%)				0.166
No	279 (64.4)	64 (58.2)	42 (54.5)	
Yes	154 (35.6)	46 (41.8)	35 (45.5)	
Gallbladder wall thickness, n (%)				0.308
<3 mm	364 (84.1)	98 (89.1)	68 (88.3)	
≥3 mm	69 (15.9)	12 (10.9)	9 (11.7)	
GBP number, n (%)				0.796
Solitary	261 (60.3)	70 (63.6)	46 (59.7)	
Multiple (≥2)	172 (39.7)	40 (36.4)	31 (40.3)	
Metabolic burden score, n (%)				<0.001
<2	198 (45.7)	42 (38.2)	18 (23.4)	
≥2	235 (54.3)	68 (61.8)	59 (76.6)	

HRA, high-risk adenoma; Non-HRA, non-high-risk adenoma; BMI, body mass index; FIB-4, fibrosis-4; GBP, gallbladder polyp; SD, standard deviation.

**Table 1B:** Distribution of the individual high-risk adenoma criteria among HRA patients.

HRA component	n (%)
Diameter ≥10 mm	27 (35.1)
Villous histological components	29 (37.7)
High-grade intraepithelial neoplasia	0 (0.0)
≥3 adenomas	53 (68.8)
Meeting ≥2 high-risk criteria*	26 (33.8)

Meeting ≥2 high-risk criteria*: patients fulfilling at least two of the four criteria.The four criteria are not mutually exclusive. Of the 77 HRA patients, 51 (66.2%) met only one criterion, 20 (26.0%) exactly two, and 6 (7.8%) three.

### 3.2 Relationship between FIB-4 category and colorectal adenoma severity

When stratified by FIB-4 index category, colorectal adenoma severity showed a clear gradient distribution (**Table 2A**, **Figure 2**). Among the 620 examinees, as the FIB-4 category increased, the proportion of no adenoma decreased from 72.0% to 66.1% and further declined to 55.0% in the high FIB-4 group. Correspondingly, the proportion of HRA showed a progressive increase, rising from 10.3% in the low category to 14.6% and reaching 40.0% in the high FIB-4 group. The proportion of non-HRA remained relatively stable in the low and intermediate FIB-4 groups (17.7%-19.3%) but decreased markedly to 5.0% in the high FIB-4 group. Further analysis using the Mantel-Haenszel linear trend test revealed a significant linear dose-response relationship between FIB-4 category and adenoma severity (χ² = 8.335, df = 1, P = 0.0039; Pearson r = 0.116), indicating that higher FIB-4 categories were associated with greater risk of colorectal adenoma, particularly HRA (**Table 2B**).

**Table 2A:** Distribution of colorectal adenoma outcomes according to FIB-4 index category.

FIB-4 index category	N	No adenoma	Non-HRA	HRA
Low (<1.3)	429	309 (72.0)	76 (17.7)	44 (10.3)
Intermediate (1.3–2.67)	171	113 (66.1)	33 (19.3)	25 (14.6)
High (>2.67)	20	11 (55.0)	1 (5.0)	8 (40.0)

**Figure 2:**
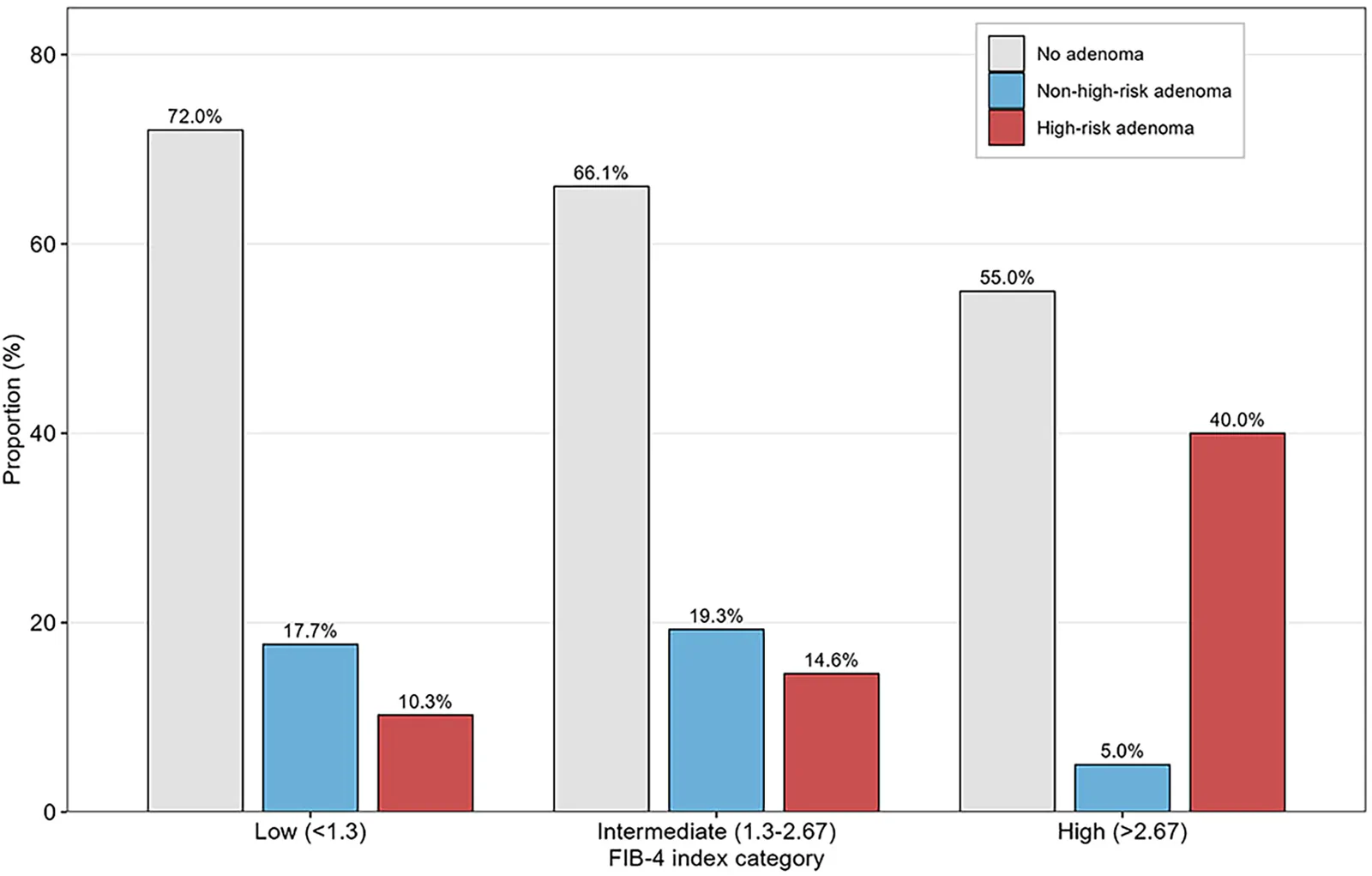
Proportions of colorectal adenoma subtypes by FIB-4 index category.

**Table 2B:** Dose-response trend of colorectal adenoma severity across FIB-4 index categories.

Test	Statistic	df	P value
Mantel-Haenszel linear trend test (χ²)	8.335	1	0.0039
Pearson correlation coefficient (r)	0.116	–	–

### 3.3 Binary logistic regression analysis of HRA

Binary logistic regression was fitted with HRA as the outcome (**Table 3**). In the unadjusted model, the high FIB-4 group (>2.67) showed a significantly elevated risk of HRA compared with the low FIB-4 group (<1.3) (OR 5.83, 95% confidence interval [CI] 2.18–14.90, P < 0.001); the intermediate FIB-4 group (1.3-2.67) did not differ significantly from the low FIB-4 group (OR 1.50, 95% CI 0.87-2.52, P = 0.132). After adjusting for age, sex, smoking, alcohol consumption, and metabolic burden score, the effect of the high FIB-4 group was substantially attenuated and no longer significant (adjusted OR [aOR] 2.32, 95% CI 0.76-6.76, P = 0.129), suggesting that the risk signal was partly confounded by these covariates. In the adjusted model, age, male sex, and metabolic burden score were independent risk factors for HRA, while smoking and alcohol consumption showed no statistically significant adjusted effects (P > 0.05). Sensitivity analysis using FIB-4 as a continuous variable yielded an aOR of 1.09 per 1-SD increase (95% CI 0.84–1.35, P = 0.41), showing no significant linear association. The ORs (and aORs) with 95% CIs for all models are detailed in **Table 3**.

**Table 3:** Crude and adjusted logistic regression for high-risk colorectal adenoma by FIB-4 index category.

Characteristics	OR (95% CI)	P	aOR (95% CI)	Adjusted P
FIB-4 index: Intermediate vs. Low	1.50 (0.87-2.52)	0.132	0.96 (0.52-1.75)	0.907
FIB-4 index: High vs. Low	5.83 (2.18-14.90)	<0.001	2.32 (0.76-6.76)	0.129
Age (years)	–	–	1.06 (1.03-1.09)	<0.001
Sex (Male vs. Female)	–	–	2.54 (1.44-4.59)	0.002
Smoking (yes vs. no)	–	–	1.35 (0.56-3.11)	0.486
Alcohol drinking (yes vs. no)	–	–	0.57 (0.19-1.52)	0.279
Metabolic burden score	–	–	1.32 (1.04-1.67)	0.022
FIB-4 per 1-SD (continuous)	–	–	1.09 (0.84-1.35)	0.41

Crude model, unadjusted; adjusted model, adjusted for age, sex, smoking, alcohol drinking, and metabolic burden score; aOR, adjusted odds ratio; CI, confidence interval; FIB-4, fibrosis-4; SD, standard deviation.

### 3.4 Subgroup analysis and effect modification

To evaluate potential effect modifiers, prespecified subgroup analyses were conducted to examine whether the association between FIB-4 and HRA varied by sex, age, metabolic burden, GBP number, and gallbladder wall thickness (**Figure 3**). FIB-4 was included as a per-1-SD increment, with HRA as the outcome. Subgroup models were fitted within each stratum, adjusting for age, sex, smoking, alcohol consumption, and metabolic burden. In the overall population, the aOR per 1-SD increase in FIB-4 for HRA was 1.09 (95% CI 0.84–1.35, P = 0.41), which did not reach statistical significance. No significant effect modification was observed across subgroups of sex (female: aOR 1.02, 95% CI 0.57–1.34; male: aOR 1.33, 95% CI 0.87-2.01), age (<51 years: aOR 0.38, 95% CI 0.10-1.16; ≥51 years: aOR 1.14, 95% CI 0.89-1.42), metabolic burden (<2 points: aOR 1.16, 95% CI 0.80-1.51; ≥2 points: aOR 1.11, 95% CI 0.72-1.66), or GBP number (solitary: aOR 1.10, 95% CI 0.81-1.38; multiple: aOR 1.10, 95% CI 0.59-2.02) (interaction P > 0.05).

**Figure 3:**
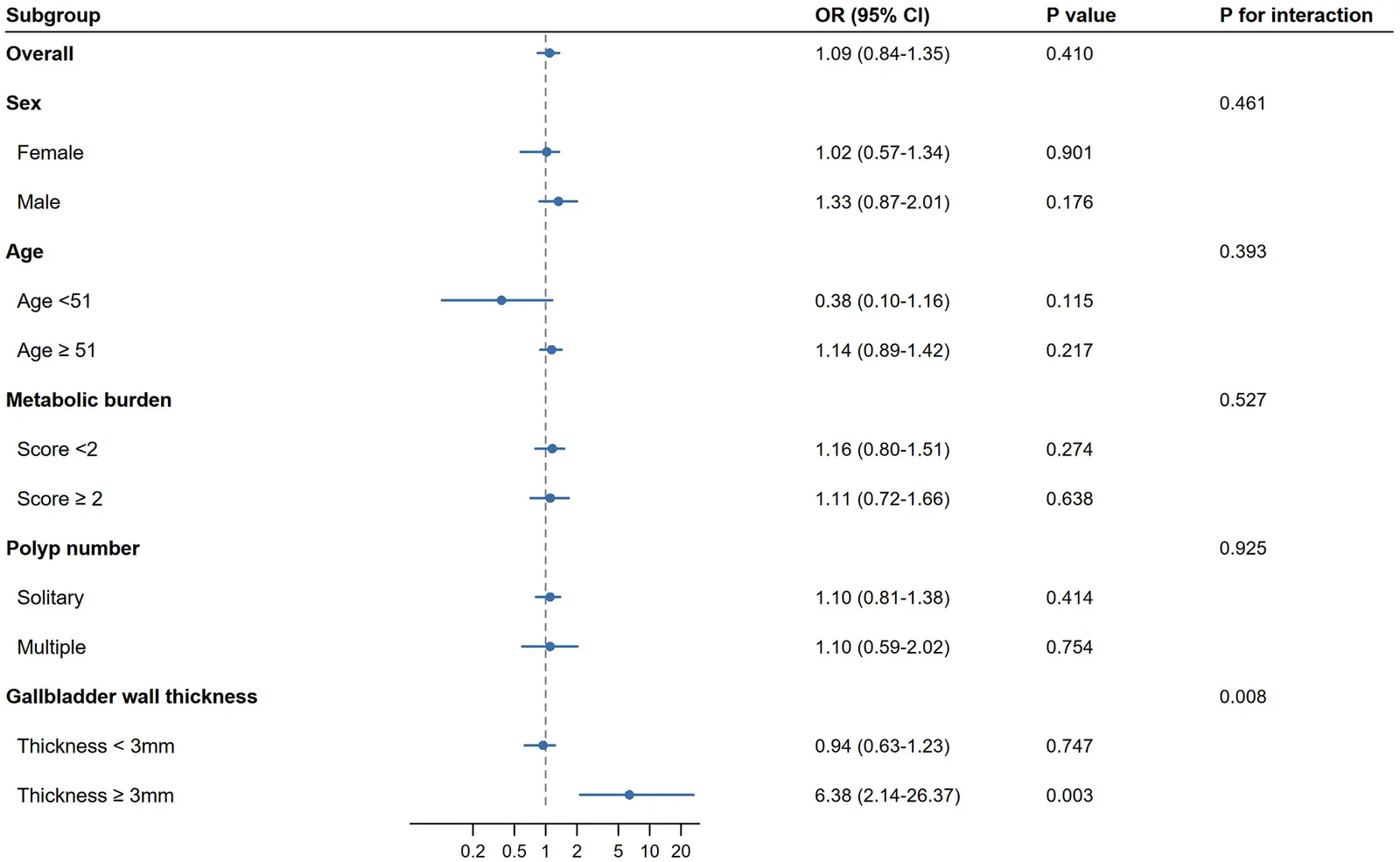
Subgroup analysis of FIB-4 (per 1-SD increase) and high-risk colorectal adenoma.

However, gallbladder wall thickness exhibited a significant effect modification on the association between FIB-4 and HRA (**Table 4**). In the gallbladder wall thickening (≥3 mm) subgroup (n = 90), the aOR per 1-SD increase in FIB-4 was 6.38 (95% CI 2.14-26.37, P = 0.003), although the wide confidence interval suggests limited precision of this estimate. In the normal gallbladder wall (<3 mm) subgroup (n = 530), this association was not statistically significant (aOR 0.94, 95% CI 0.63-1.23, P = 0.75). The multiplicative interaction test confirmed the statistical significance of this interaction (Wald P = 0.008, LRT P = 0.001), indicating that gallbladder wall thickness significantly enhances the association between FIB-4 and HRA in patients with GBPs. Owing to the small number of events in this subgroup, the conventional multivariable model was at risk of overfitting; accordingly, robustness analyses were further performed using Firth penalized logistic regression and a simplified model adjusting only for age and sex (**Table 5**). Across the three models (conventional, Firth penalized, and simplified), the adjusted OR per 1-SD increase in FIB-4 was 6.38, 4.17, and 6.40, respectively, and none of the 95% CIs crossed 1. In the Firth penalized model, which corrects for small-sample bias, the point estimate shrank from 6.38 to 4.17, suggesting that the association in this subgroup is relatively insensitive to model specification. Although the aforementioned subgroup analyses and multiplicative interaction tests were prespecified, because no correction for multiple comparisons was applied, this significant interaction should still be interpreted cautiously as an exploratory, hypothesis-generating finding and requires further validation in independent external cohorts.

**Table 4:** Interaction between FIB-4 index and gallbladder wall thickness for high-risk adenoma.

Parameter	β (SE)	OR (95% CI)	P value
FIB-4(per 1-SD increase) × gallbladder wall thickness	1.39 (0.53)	4.03 (1.62-13.47)	0.008
Likelihood ratio test	–	–	0.001

Gallbladder wall thickness categorized as ≥3 mm or <3 mm; model adjusted for age, sex, smoking, drinking, metabolic burden.

**Table 5:** Robustness analysis of the association between FIB-4 (per 1-SD increase) and high-risk adenoma in the gallbladder wall-thickening subgroup.

Model	Adjusted variables	aOR (95% CI)	P value
Conventional multivariable logistic	age, sex, smoking, drinking, metabolic burden score	6.38 (2.14-26.37)	0.003
Firth-penalized logistic	age, sex, smoking, drinking, metabolic burden score	4.17 (1.68-14.59)	0.001
Simplified logistic	age, sex	6.40 (2.20-25.90)	0.003

All three models report profile-likelihood 95% CIs; the Firth-penalized model additionally applies a Jeffreys prior; aOR, adjusted odds ratio.

## 4 Discussion

This study found a dose-response relationship between FIB-4 category and colorectal adenoma severity in patients with GBPs (linear trend P = 0.0039), but this association was attenuated after adjustment for demographic and metabolic factors, suggesting partial confounding. Among these adjusted covariates, metabolic burden score, age, and male sex were independent factors associated with HRA in patients with GBPs, consistent with the direction of prior population-level evidence. Cheng et al. (19) found that age, male sex, smoking, and metabolic syndrome were risk factors for advanced colorectal neoplasia in fecal immunochemical test-negative populations. Azimi et al. (20), in a systematic review and meta-analysis of over 56 million individuals, also demonstrated that metabolic-associated fatty liver disease is associated with an increased risk of colorectal polyps and neoplasia. Regarding liver fibrosis itself, Kim et al. (21) reported that in patients with nonalcoholic fatty liver disease, the degree of liver fibrosis is associated with colorectal adenoma risk, suggesting that fibrosis burden may not merely be an epiphenomenon of metabolic dysfunction. FIB-4 is calculated from age, AST, ALT, and PLT; given that age and metabolic burden were themselves independent correlates of HRA in this study, the attenuation of the FIB-4 effect after adjustment suggests that its risk signal may originate primarily from these included covariates rather than an independent fibrosis-related effect. Limited by the cross-sectional design, this study cannot establish the temporal relationship between liver fibrosis reflected by FIB-4 and HRA detection, precluding causal inference. Furthermore, the high FIB-4 group comprised only 20 patients, yielding a post-adjustment 95% CI of 0.76–6.76; this wide interval indicates limited precision, and the negative result should not be equated with confirmation of no association.

Notably, this study found that gallbladder wall thickness on ultrasound exhibited a significant effect modification on the association between FIB-4 and HRA in patients with GBPs. Among patients with gallbladder wall thickening (≥3 mm), each 1-SD increase in FIB-4 was significantly associated with HRA (aOR = 6.38, P = 0.003), with the interaction reaching significance by both the Wald test (P = 0.008) and the LRT (P = 0.001), whereas no such association was observed in patients with normal wall thickness (aOR = 0.94, P = 0.75). Gallbladder wall thickening detected on abdominal ultrasound most often indicates chronic gallbladder inflammation (22). To objectively quantify the severity of gallbladder inflammation, Madni et al. (23) proposed the Parkland grading scale (grades I–V), which stratifies the degree of inflammation according to intraoperative findings and has been validated in an independent cohort to predict the difficulty and outcomes of laparoscopic cholecystectomy (24). Building on this, Uçaner et al. (25) demonstrated that the proportion of patients with a preoperative ultrasound gallbladder wall thickness >4 mm increased progressively with higher Parkland grade, and that those with wall thickening had longer operative times and higher rates of perforation and conversion to open surgery. It should be noted that Uçaner et al. (25) studied a population of cholecystectomy patients and used a preoperative ultrasound gallbladder wall thickness >4 mm as the cutoff, whereas the present study focused on patients with gallbladder polyps and defined wall thickening as a fasting wall thickness ≥3 mm; thus, the two studies differ in both population characteristics and the wall-thickness cutoff applied. Nevertheless, Uçaner et al. (25) confirmed that wall thickness is associated with the severity of gallbladder inflammation, providing external support for the rationale of treating wall thickening as an effect modifier in the present study. These findings suggest that gallbladder wall thickness on ultrasound reflects the severity of gallbladder disease. In light of the above evidence, the aforementioned effect-modification finding of this study suggests that the risk stratification performance of FIB-4 is not uniform within the GBP population but may depend on the local pathological state of the gallbladder. When a single serological index is interpreted in isolation without imaging context, the stratification information it carries may be substantially diluted. This direction is consistent with existing practice in risk stratification of precancerous lesions: Gupta et al. (26) showed that integrating routinely available clinical characteristics can further distinguish risk strata within adenoma populations; Yoo et al. (27) also reported that skeletal muscle mass, an easily measurable body composition indicator, is associated with the risk of advanced adenoma at surveillance colonoscopy. It must be emphasized that this study did not construct or validate a prediction model; the screening value of FIB-4 combined with gallbladder wall thickness on ultrasound remains a risk stratification hypothesis supported by interaction testing and awaits validation in independent cohorts.

The epidemiological link between gallbladder disease and colorectal neoplasia may be related to bile acid metabolism and the gut-liver axis. Wu et al. (28) observed altered plasma bile acid profiles in patients with gallbladder stones and polyps. Cong et al. (29) demonstrated that bile acids modified by the intestinal microbiota can suppress CD8^+^ T cell effector functions, thereby promoting colorectal cancer growth. Tang et al. (30) further showed that gut microbiota dysbiosis following cholecystectomy can exacerbate colorectal tumorigenesis. Fuchs et al. (31) and Wang et al. (32) each systematically described the pathophysiological frameworks of bile acid metabolism signaling networks and the gut-liver axis, respectively. The above evidence collectively outlines a potential pathway of gallbladder disease, bile acid dysregulation, gut microbiota, and colorectal mucosal changes, providing a contextual explanation for the enhanced association observed in the gallbladder wall thickening subgroup. Regarding a potential direct link between FIB-4 and gallbladder disease, Dong et al. (33), based on NHANES 2017-2020, observed a nonlinear positive association between FIB-4 and gallstone prevalence; however, that study used gallstones rather than GBPs as the outcome and was also cross-sectional in design, serving only as indirect circumstantial evidence that liver fibrosis indicators and gallbladder disease tend to co-occur in populations enriched for metabolic risk factors. It should be noted that this study did not measure bile acid profiles or gut microbiota; the above pathway remains an explanatory hypothesis requiring direct verification in future studies.

From a clinical accessibility perspective, both indicators used in this study are simple, noninvasive, and readily available: FIB-4 can be calculated from routine blood test results, and abdominal ultrasound is widely performed even in primary care hospitals. Accordingly, the combined application of FIB-4 and gallbladder ultrasound characteristics is practically feasible. In particular, among patients with GBPs accompanied by gallbladder wall thickening, elevated FIB-4 signifies a higher risk of HRA, offering clues for optimizing colonoscopy screening stratification in this subgroup. However, this study still has certain limitations. First, there is selection bias: among the 13, 315 patients with GBPs initially screened, only 882 (approximately 6.6%) underwent colonoscopy, and 620 were included in the analysis after applying exclusion criteria. The colonoscopies were performed based on clinical context rather than systematic screening, and the examined individuals were not a representative sample of the GBP population; therefore, the results cannot be generalized to all patients with GBPs. Second, the metabolic burden score in this study is a nonstandard composite indicator simplified from the metabolic syndrome framework—it substitutes fatty liver for waist circumference, and the equal weighting of 1 point per component is an arbitrary setting; as a covariate, its control of metabolic confounding may be incomplete, and the relevant conclusions should be interpreted with caution and require further validation. Third, gallbladder wall thickening was defined as a fasting wall thickness ≥3 mm, and the data were retrospectively extracted from the Picture Archiving and Communication System; potential inter-observer variability in interpretation may exist among different reporters, and this assessment awaits further validation through prospective standardized measurements. Furthermore, this study is limited by its single-center cross-sectional design, and the subgroup sample on which the interaction is based is limited. The optimal cutoff for combined screening and its incremental value over conventional risk factors require confirmation in multicenter prospective cohorts.

## 5 Conclusion

Gallbladder wall thickening (≥3 mm) is a key effect modifier of the FIB-4-HRA association: only in this subgroup was each 1-SD increase in FIB-4 significantly associated with HRA (aOR 6.38, P = 0.003), and the multiplicative interaction term was also significant (Wald P = 0.008, LRT P = 0.001). Therefore, in patients with GBPs and gallbladder wall thickening, elevated FIB-4 may indicate an increased risk of HRA, offering clues for optimizing colonoscopy screening stratification in this subgroup.

## Data Availability

The original contributions presented in the study are included in the article/supplementary material. Further inquiries can be directed to the corresponding author.
